# T111. PANSS NEGATIVE SYMPTOM DIMENSIONS ACROSS GEOGRAPHICAL REGIONS: IMPLICATIONS FOR SOCIAL, LINGUISTIC AND CULTURAL CONSISTENCY

**DOI:** 10.1093/schbul/sby016.387

**Published:** 2018-04-01

**Authors:** Anzalee Khan, Lora Liharska, Philip Harvey, Alexandra Atkins, Richard Keefe, Danny Ulshen

**Affiliations:** 1 Nathan S. Kline Institute for Psychiatric Research; 2 New York University School of Medicine; 3 Leonard M. Miller School of Medicine, University of Miami; 4 NeuroCog Trials; 5 Duke University Medical Center

## Abstract

**Background:**

Recognizing the discrete dimensions that underlie negative symptoms in schizophrenia and how these dimensions are conceptualized across geographical regions may result in better understanding and treatment. The expressive-experiential distinction has been shown to have vast importance in relation to functional outcomes in schizophrenia. Previous studies have shown that the PANSS may not be equivalently rated across counties and cultures, suggesting regional differences in both symptom expression and rater judgment of symptom severity. Items that perform in markedly different ways across demographic, regional, cultural, or clinical severity characteristics may not offer valid representations of the target construct. 1) Will the expressive and experiential dimensions of the PANSS vary over 15 geographical regions and will the item ratings defining each dimension manifest similar reliability across these regions? 2) In large multi-center, international trials where data are combined, which of the two dimensions are disposed to social, linguistic and cultural inconsistency?

**Methods:**

Data was obtained for the baseline PANSS visits of 6,889 subjects. Using Confirmatory Factor Analysis (CFA), we examined whether the expressive-experiential distinction would be replicated in our sample. We investigated the validity of the expressive-experiential distinction using Differential Item Functioning (DIF; Mantel-Haenszel) across 15 geographical regions – South America-Mexico, Austria-Germany, Belgium-Netherlands, Brazil, Canada, Nordic regions (Denmark, Finland, Norway, Sweden), France, Great Britain, India, Italy, Poland, Eastern Europe (Romania, Slovakia, Ukraine, Croatia, Estonia, Czech Republic), Russia, South Africa, and Spain - as compared to the United States.

**Results:**

Expressive Deficit: More DIF was observed for items in the Expressive deficit factor than for items relating to experiential deficits. The following regions showed at least moderate to large DIF for all items: Austria-Germany, Nordic, France, and Poland. Of all the items, N3 Poor Rapport showed the most moderate and large DIF (n = 13; 86.67%) across countries, with 7 countries reporting large DIF. Similarly, N6 Lack of Spontaneity and Flow of Conversation showed moderate and large DIF for 66.67% countries (n=10). Experiential Deficit: Item G16 Active Social Avoidance reported negligible DIF for 14 of the 15 countries investigated (93.33%). Large DIF was observed for N2 Emotional Withdrawal and N4 Passive Apathetic Social Withdrawal for Brazil and India. Seven regions demonstrated no DIF across all items of the PANSS experiential deficit factor (South America-Mexico, Belgium-Netherlands, Nordic, Great Britain, Eastern Europe, Russia, and Spain). Overall, there were many fewer observed items with large DIF for PANSS experiential domain.

**Discussion:**

These results suggest that the PANSS Negative Symptoms Factor can be better represented by a two-factor model than by a single-factor model. Additionally, the results show significant differences in ratings on the PANSS expressive items, but not the experiential items, across regions. This could be due to a lack of equivalence between the original and translated versions, cultural differences in the interpretation of items, rater training, or understanding of scoring anchors. Knowing which items are challenging for raters across regions can help guide PANSS training to improve results of international clinical trials aimed at negative symptoms.

